# Community structure of partial nitritation‐anammox biofilms at decreasing substrate concentrations and low temperature

**DOI:** 10.1111/1751-7915.12435

**Published:** 2016-11-14

**Authors:** Frank Persson, Carolina Suarez, Malte Hermansson, Elzbieta Plaza, Razia Sultana, Britt‐Marie Wilén

**Affiliations:** ^1^ Division of Water Environment TechnologyDepartment of Civil and Environmental EngineeringChalmers University of TechnologySE‐41296GothenburgSweden; ^2^ Department of Chemistry and Molecular BiologyUniversity of GothenburgSE‐40530GothenburgSweden; ^3^ Department of Sustainable DevelopmentEnvironmental Science and Engineering (SEED)Royal Institute of Technology (KTH)Teknikringen 76SE‐100 44StockholmSweden

## Abstract

Partial nitritation‐anammox (PNA) permits energy effective nitrogen removal. Today PNA is used for treatment of concentrated and warm side streams at wastewater treatment plants, but not the more diluted and colder main stream. To implement PNA in the main stream, better knowledge about microbial communities at the typical environmental conditions is necessary. In order to investigate the response of PNA microbial communities to decreasing substrate availability, we have operated a moving bed biofilm reactor (MBBR) at decreasing reactor concentrations (311–27 mg‐N l^−1^ of ammonium) and low temperature (13°C) for 302 days and investigated the biofilm community using high throughput amplicon sequencing; quantitative PCR; and fluorescence *in situ* hybridization. The anammox bacteria (*Ca*. Brocadia) constituted a large fraction of the biomass with fewer aerobic ammonia oxidizing bacteria (AOB) and even less nitrite oxidizing bacteria (NOB;* Nitrotoga*,* Nitrospira* and *Nitrobacter*). Still, NOB had considerable impact on the process performance. The anammox bacteria, AOB and NOB all harboured more than one population, indicating some diversity, and the heterotrophic bacterial community was diverse (seven phyla). Despite the downshifts in substrate availability, changes in the relative abundance and composition of anammox bacteria, AOB and NOB were small and also the heterotrophic community showed little changes in composition. This indicates stability of PNA MBBR communities towards decreasing substrate availability and suggests that even heterotrophic bacteria are integral components of these communities.

## Introduction

Autotrophic nitrogen removal from wastewater can be achieved by partial nitritation together with anaerobic ammonium oxidation (anammox). Partial nitritation‐anammox (PNA) saves energy due to a reduced need for aeration by > 50% (Siegrist *et al*., 2008) and enables a higher utilization of organic carbon for production of valuable products, for example, biogas, compared to conventional nitrogen removal with nitrification‐denitrification. Together, this makes energy positive wastewater treatment plants (WWTPs) possible (Kartal *et al*., 2010).

Today, PNA is established for treatment of warm and concentrated wastewater side streams (Lackner *et al*., 2014), where the conditions for growth of aerobic ammonia oxidizing bacteria (AOB) and anammox bacteria are beneficial and inhibition of unwanted aerobic nitrite oxidation by nitrite oxidizing bacteria (NOB) can be effective. However, the side stream nitrogen removal at WWTPs treats only 15–20% of the total nitrogen. To utilize the benefits of PNA in the wastewater main stream is highly desirable and has recently become a prioritized research area. In the main stream, the conditions for PNA are much more challenging (De Clippeleir *et al*., 2013; Hu *et al*., 2013; Laureni *et al*., 2016). The cold and diluted water causes low activity and slow growth rate of particularly the anammox bacteria (Hendrickx *et al*., 2014; Lotti *et al*., 2015), even though some adaptations to low temperatures have been observed (Dosta *et al*., 2008; Hu *et al*. 2013; Hendrickx *et al*., 2014). Moreover, the competition between AOB and anammox bacteria with NOB and denitrifying bacteria is challenging at these conditions (see e.g. De Clippeleir *et al*., 2013; Perez *et al*., 2014), which necessitates detailed knowledge about the dynamics of these microorganisms in order to understand process performance.

Despite the challenges, maintenance and activity of AOB and anammox bacteria at low temperatures and low substrate concentrations have been demonstrated in biofilm‐ and granular sludge reactors (De Clippeleir *et al*., 2013; Gustavsson *et al*., 2014; Lotti *et al*., 2014a; Gilbert *et al*., 2015; Ma *et al*., 2015; Laureni *et al*., 2016) and in a few studies the major population of AOB, anammox bacteria and NOB have been identified (Gilbert *et al*., 2014; Lotti *et al*., 2014a). Little is, however, known about the microbial community structure and dynamics at main stream conditions (Gilbert *et al*., 2014) and how such communities differ from the communities in the PNA reactors treating concentrated wastewater with higher substrate availability. Furthermore, the heterotrophic bacteria in PNA reactors (Gilbert *et al*., 2014; Pellicer‐Nàcher *et al*., 2014; Chu *et al*., 2015) most likely affect the nitrogen turnover and process performance, but the composition, diversity and roles of these are little investigated, particularly at main stream conditions.

Here, an experiment was designed to investigate the role of the substrate concentration in shaping the PNA microbial community in a MBBR over 302 days. The influent nitrogen concentration was decreased from 500 to 45 mg‐N l^−1^ at low temperature (13°C) to stepwise approach main stream conditions. For investigation of the composition and diversity of the total bacterial community (including heterotrophic bacteria), the abundance of key functional groups and their localization in the biofilms, a multiphase approach of high throughput amplicon sequencing (Illumina MiSeq), quantitative PCR (qPCR) and fluorescence *in situ* hybridization (FISH) in conjunction with confocal laser scanning microscopy (CLSM) of cryosectioned biofilms was used. Reactor performance and potential activity of key functional groups was also monitored.

## Results

### Reactor performance

The concentration of the influent was decreased from target concentrations of 500 to 45 mg‐N l^−1^ from period I to period VI (Figure S1) resulting in average ammonium concentrations of 311 to 27 mg‐N l^−1^ in the reactor. During periods I to V, the nitrogen removal rate (NRR) was rather similar, with a decrease in period VI, at the lowest influent ammonium concentration (Table 1). The biomass weight on the carriers was stable, with insignificant changes during the study period (Figure S3, ANOVA, *P* > 0.05). The set‐up and the performance of the reactor is summarized in Table 1. Time‐course displays of nitrogen species are found in Figure S1.

**Table 1:** Study design and operational data of the MBBR

	Period I	Period II	Period III	Period IV	Period V	Period VI
*Operational set‐up*						
Time (d)	1–55	62–99	106–133	136–189	195–258	262–302
NH_4_‐N _infl_ (mg l^−1^)	496 ± 32	249 ± 14	170 ± 19	129 ± 11	86 ± 8	43 ± 2
COD _infl_ (mg l^−1^)	313 ± 36	174 ± 7	155 ± 26	84 ± 9	56 ± 4	48 ± 8
HRT (d)	3.3 ± 0.2	3.2 ± 0.0	3.2 ± 0.0	2.3 ± 0.1	1.6 ± 0.0	0.8 ± 0.0
NLR (g‐N m^‐2^ d^−1^)	0.75 ± 0.03	0.39 ± 0.02	0.26 ± 0.03	0.29 ± 0.02	0.28 ± 0.03	0.26 ± 0.02
pH	7.9 ± 0.12	7.7 ± 0.09	7.8 ± 0.08	7.5 ± 0.19	7.1 ± 0.07	7.2 ± 0.12
DO (mg l^−1^)	0.93 ± 0.08	0.67 ± 0.04	0.64 ± 0.07	0.82 ± 0.25	0.49 ± 0.06	0.48 ± 0.10
*Reactor performance*						
NH_4_‐N _effl_ (mg l^−1^)	311 ± 70	136 ± 21	119 ± 12	63 ± 22	31 ± 10	27 ± 5
NO_2_‐N _effl_ (mg l^−1^)	12.5 ± 5.6	6.0 ± 5.7	2.2 ± 1.0	2.8 ± 1.4	1.6 ± 0.7	1.8 ± 0.3
NO_3_‐N _effl_ (mg l^−1^)	98 ± 41	33 ± 23	8 ± 4	17 ± 13	21 ± 7	9 ± 6
COD _effl_ (mg l^−1^)	281 ± 15	153 ± 19	118 ± 8	67 ± 14	53 ± 8	40 ± 6
ARR (g‐N m^−2^ d^−1^)	0.28 ± 0.10	0.18 ± 0.04	0.11 ± 0.07	0.14 ± 0.05	0.18 ± 0.04	0.10 ± 0.04
NRR (g‐N m^−2^ d^−1^)	0.11 ± 0.06	0.12 ± 0.03	0.09 ± 0.07	0.10 ± 0.03	0.10 ± 0.03	0.03 ± 0.03
NO_3_ production (%)	51 ± 19	28 ± 16	15 ± 8	23 ± 11	35 ± 12	58 ± 26

HRT = hydraulic retention time, NLR = nitrogen loading rate, DO = dissolved oxygen, ARR = ammonia removal rate, NRR = nitrogen removal rate. COD and nitrogen species are dissolved (filtered, 0.45 μm).

### Abundance of autotrophic nitrogen converting bacteria

**Figure 1:**
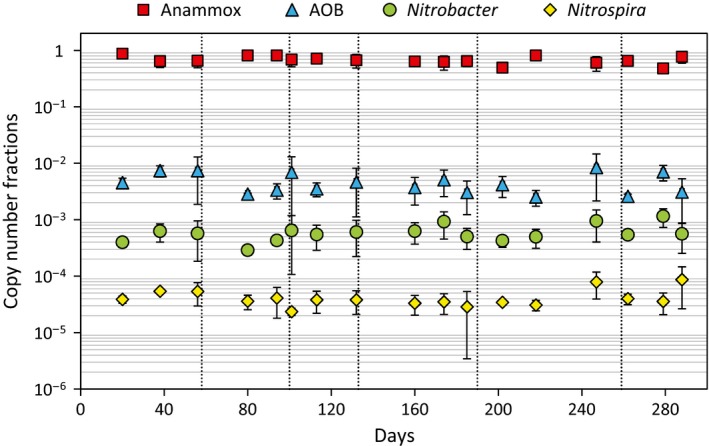
Relative abundances (copy number fractions) of nitrogen converting bacteria measured by qPCR. The periods in the study are separated by vertical dashed lines. Average values. Error bars show standard deviation.

The anammox bacteria dominated the total bacterial community in all periods, as measured by qPCR (Fig. 1). The gene copy numbers of AOB (amoA) were about two orders of magnitude lower than the anammox bacteria (16S rRNA). The abundances of the NOB, *Nitrobacter* and *Nitrospira*, were even lower. There were no major changes in the relative abundances of the anammox bacteria, AOB, *Nitrobacter* and *Nitrospira* over the course of the study, as measured by qPCR (ANOVA, *P* > 0.05), and correlations between the relative abundances for each sampling occasion (*n* = 17) and the reactor concentrations of nitrogen species, COD and alkalinity were non‐significant (*P* > 0.05). The methods of qPCR, FISH and amplicon sequencing, all showed that anammox bacteria was the largest group followed by AOB and NOB (Fig. 1 and Table S4). FISH detected a higher percentage of AOB than the other methods, but it should be noted that only 20 to 40% of the cells detected by a general DNA stain (SYTO62) were detected by the general FISH EUB probe mix (Figure S4).

### Batch activity tests of nitrogen converting groups of microorganisms

**Figure 2:**
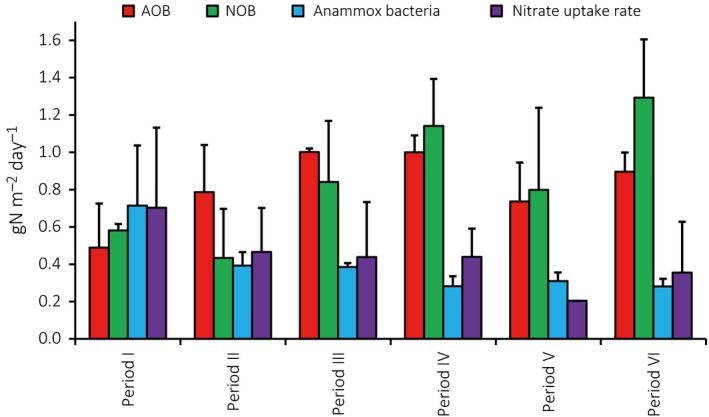
Potential activity of nitrogen converting bacterial guilds measured in batch tests at period I–VI. Bars show average values, error bars show standard deviation.

Batch tests (Fig. 2) were performed to assess the potential aerobic ammonium oxidation (AOB), aerobic nitrite oxidation (NOB), anammox and nitrate uptake rate (potential denitrification). Although the variations between samples and sampling occasions were considerable, the data showed a gradual decrease in potential anammox (ANOVA, *P* < 0.01, *F* = 5.7) and an increase in potential nitrite oxidation (ANOVA, *P* < 0.01, *F* = 4.6). For the aerobic ammonium oxidation and the nitrate uptake rate, the changes between the periods were not significant (*P* > 0.05).

### Composition and diversity of the biofilm communities

High throughput amplicon sequencing of the 16S rRNA gene (V4 region) showed that the bacterial communities at four periods in the MBBR consisted of similar numbers of OTUs (477 to 523), when resampled at 10 000 sequences (Table S2) and of all the 886 OTUs detected, 236 were shared by all samples. The diversity of the sample from the final period (VI) was somewhat higher, indicative of a slightly more even community (Table S2). Pairwise comparisons of the biofilm communities (Bray‐Curtis) in periods I, III and V resulted in coefficients of 0.10–0.11, while period VI differed slightly more (0.15–0.18). However, the dissimilarity coefficients were generally low, indicating similar community structure of all samples.

**Figure 3:**
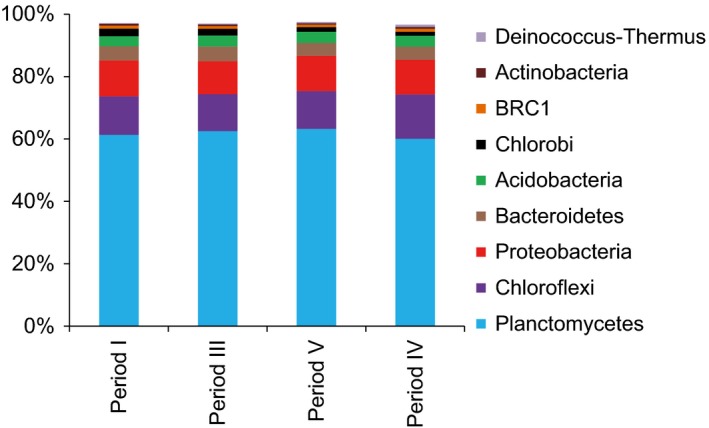
Major bacterial phyla (> 0.5% relative sequence contribution) in the biofilm communities, as revealed by high throughput amplicon sequencing.

The composition at the phylum level showed that the majority of the sequences belonged to *Planctomycetes*, with *Chloroflexi* and *Proteobacteria* also having large contributions to the community (Fig. 3). In addition, *Bacteroidetes*,* Acidobacteria*,* Chlorobi*, the candidate phylum BRC1, *Actinobacteria* and *Deinococcus‐Thermus* were present in relative sequence abundances > 0.5%.

The anammox bacteria were all affiliated to the genus *Ca*. Brocadia in one single OTU that dominated the bacterial biofilm community in all reactor periods (Table 2). Further subdivision of the sequences within *Ca*. Brocadia, by applying a more stringent criterion of 99% sequence similarity of OTUs, revealed that most anammox bacteria were similar to *Ca*. Brocadia sp. 40, with a smaller population of *Ca*. Brocadia fulgida‐like bacteria (Table S3). This population was also detected by FISH (0.5–2.0% of total bacteria).

**Table 2:** Autotrophic nitrogen converting bacteria in the biofilm communities, as revealed by high throughput amplicon sequencing. OTUs clustered at 97% sequence similarity. BLAST analysis was used for classification

OTU	Classification	Similarity	Period I	Period III	Period V	Period IV
2	*Ca*. Brocadia sp. 40/*Ca*. B. caroliniensis 20b	98	56%	56%	57%	50%
85	*Nitrosomonas europaea*/*N. eutropha*	99	0.32%	0.27%	0.14%	0.25%
3	*Nitrosomonas* sp. JL21	100	0.038%	0.026%	0.032%	0.014%
2049	*Nitrosospira multiformis*	97	0.017%	0.010%	0.012%	0.028%
930	*Ca*. Nitrotoga sp. clone JS16NT08	100	0.13%	0.051%	0.071%	0.047%
354	*Nitrospirales* 4‐29^a^	N.A.	0.11%	0.18%	0.059%	0.079%

a No described species with > 90% similarity from BLAST analysis. Classification by the Greengenes taxonomy.

All identified AOB were affiliated to *Betaproteobacteria* and the major OTU (OTU 85) was affiliated to the *Nitrosomonas europaea/eutropha* cluster (Table 2). Separate populations of *Nitrosomonas europaea* and *Nitrosomonas eutropha* within this cluster could be detected at 99% sequence similarity (Table S3). OTUs similar to *Nitrosomonas* sp. JL21 and *Nitrosospira multiformis* were also observed (Table 2), but at very low relative abundances. The presence of *Nitrosomonas europaea/eutropha*,* Nitrosomonas oligotropha* (JL21) and *Nitrosospira* was confirmed by FISH (Fig. 5) at relative abundances of 2.4–6.7%, 0–0.2% and 0.06–0.6% respectively.

Among the NOB, one OTU belonged to the order *Nitrospirales* (Table 2) but could not be affiliated to any described bacterium. However, BLAST analysis showed high similarities to unclassified sequences from other nitrogen converting wastewater reactors (OTU1534‐1535 in Table S3), suggesting that these non‐described *Nitrospirales* converted nitrogen. In addition, the presence of *Nitrospira* was demonstrated by qPCR and FISH (Table S4), but no FISH signal was seen with comammox probes for *Nitrospira nitrosa* and *N. nitrificans* (Table S1). One OTU highly similar to *Nitrotoga* was detected in all biofilm communities (Table 2) and *Nitrotoga* cells were also detected by FISH (Table S4). No OTU was assigned to *Nitrobacter*, although *Nitrobacter* was detected by qPCR and FISH (Table S4). However, OTUs with high similarity to *Nitrobacter* sp., but also to other species within *Bradyrhizobiaceae*, were revealed by BLAST (OTU 0176, 0226 in Table S3). Hence, the sequence information in the V4 region of the 16S rRNA gene was not sufficient for *Nitrobacter* identification.

**Figure 4:**
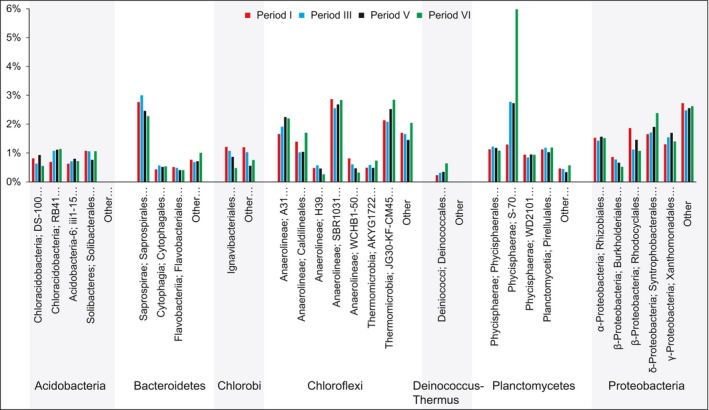
Major orders (> 0.5% relative sequence contribution) of bacteria not involved in autotrophic nitrogen conversion in the biofilm communities, as revealed by high throughput amplicon sequencing.

In the MBBR, 43–50% of the sequences were affiliated to putative heterotrophic bacteria. These bacteria were subdivided into 25 orders with a sequence contribution > 0.5% in any of the samples (Fig. 4).

### Localization of key bacterial groups in the biofilm

**Figure 5:**
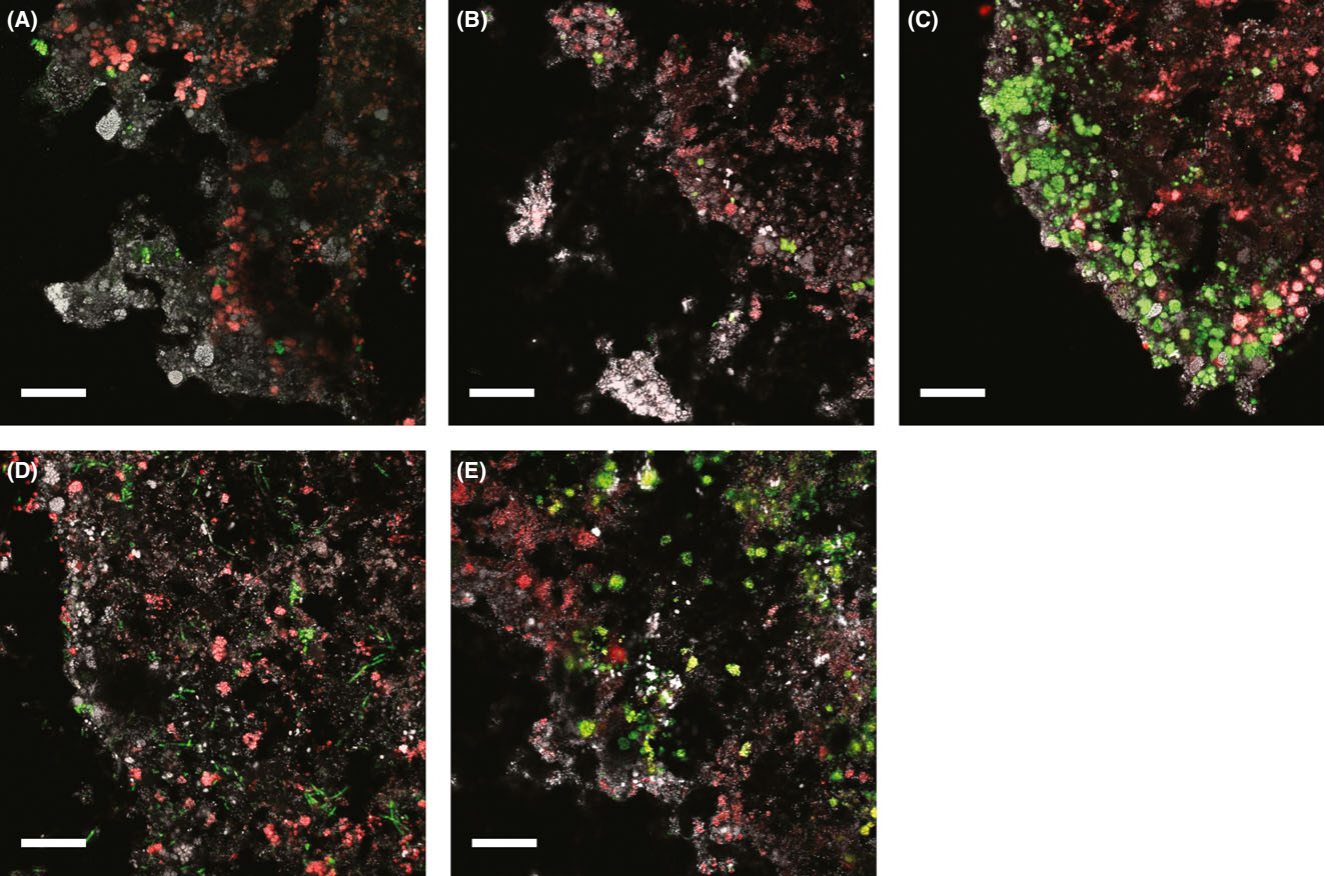
FISH‐CLSM of biofilm cryosections. The water‐biofilm interface is oriented to the lower left. In all images, anammox bacteria (Amx820) are in red and nonspecific bacteria (EUB‐mix) are in white‐grey. A. In green: AOB within the *Nitrosomonas europaea/eutropha* cluster (Nse1472). B. In green: AOB within *Nitrosospira* (Nsv443). C. In green: NOB within *Nitrospira* (Ntspa662). D. In green: Bacteria within *Chloroflexi* (CFX123 + GNSB941). E. In green: *Ca*. Brocadia fulgida (Bfu613). Scale bar: 25 μm.

FISH‐CLSM of biofilm cryosections was used to show the localization of key bacterial groups (for FISH probes, see Table S1). Clusters of AOB (*Nitrosomonas europaea/eutropha*, Fig. 5A) and *Nitrosospira*, Fig. 5B), and clusters of NOB (*Nitrospira*, Fig. 5C) were detected near the biofilm–water interface. Anammox bacteria were observed in high numbers deeper in the biofilm (Fig. 5), with two populations present closer to the biofilm–water interface (Fig. 5E). Bacteria within the phylum *Chloroflexi* were also detected both near the biofilm–water interface and deeper in the biofilm (Fig. 5D).

## Discussion

Substrate availability is a main factor that determines microbial competitive interactions and thereby shapes the structure of microbial communities (Hibbing *et al*., 2010; Litchman *et al*., 2015). PNA is used for treatment of highly concentrated as well as diluted streams of wastewater and the substrate concentrations vary a lot (e.g. Hu *et al*., 2013; Lackner *et al*., 2014; Lotti *et al*., 2014a), but very few systematic studies of the community response to changes in substrate availability have been made. Here, we test the hypothesis that a reduction in substrate availability influences the PNA microbial community structure and function in a MBBR at low temperature, gradually approaching main stream conditions. The stepwise diluted influent resulted in decreased reactor ammonium concentrations, from 311 to 27 mg‐N l^−1^, as well as decreased concentrations of COD (Table 1). Despite these decreases in substrate concentrations, no major effects on the reactor biomass were observed. The biofilm weight did not change significantly (Table S3). The anammox bacteria dominated the bacterial community with AOB and NOB being considerably fewer (Fig. 1, Table 2, S4), located near the biofilm–water interface (Fig. 5). Changes in the relative abundances of anammox bacteria, AOB and NOB (qPCR) were non‐significant between test periods and could not be related to the reactor concentrations of nitrogen species, COD and alkalinity. Changes in potential activity of the anammox bacteria and NOB were observed (Fig. 2) and reflected the nitrogen conversions in the reactor (Table 1), but in general reactor operation was stable (Table 1), suggesting similar functionality of the microbial community.

The estimation of the relative abundances of anammox bacteria, AOB and NOB generally agreed between the methods (Table S4). The largest deviation was the higher AOB percentage assessed by FISH, but smaller differences among the methods were observed also for anammox bacteria and NOB. Discrepancies between rRNA‐based FISH and DNA‐based qPCR and sequencing can be expected. A large fraction of the microbial community had a ribosomal content below the FISH detection limit (Figure S4). However, AOB are known to retain their ribosomes even at challenging conditions such as starvation (Morgenroth *et al*., 2000), which may help to explain their particularly high relative abundance observed by FISH. Also, the DNA extraction methodology, the choice of PCR primers and the cell copy numbers of target genes influence relative abundances (see e.g. Acinas *et al*., 2004; Albertsen *et al*., 2015). Quantification by high throughput amplicon sequencing is furthermore technically challenging (Zhou *et al*., 2015). Although sequence processing has improved significantly with pipelines, such as Mothur used here (Kozich *et al*., 2013), relative abundances, especially of rare OTUs (e.g. the NOB), have to be interpreted with caution. Hence, all methods suffer from limitations and multiple methods provide important complementary information. The methods also vary in response time. Specific populations can be estimated by qPCR and FISH on suspended biofilms within a day or two, which is useful for routine monitoring. High throughput amplicon sequencing and FISH biofilms cryosections provide more detailed information, but takes considerably longer time.

The major anammox population was highly similar to *Ca*. Brocadia sp. 40 (Table 2, Table S3), which has previously been observed in several anammox reactors (van der Star *et al*., 2008; Park *et al*., 2010; Costa *et al*., 2014; Gilbert *et al*., 2014). Only one population of anammox bacteria is usually observed in PNA reactors (Hu *et al*., 2013; Gilbert *et al*., 2014; Laureni *et al*., 2015). However, using 99% similarity for sequence clustering (Table S3) and a competitor probe to improve the FISH specificity (Table S1, Persson *et al*. (2014)), a second, closely related, *Ca*. Brocadia fulgida‐like population was detected (Fig. 5, Table S3). It is likely that the two populations have different niches, just as described for closely related *Nitrospira* strains (Gruber‐Dorninger *et al*., 2014). This was supported by the localization of the smaller *Ca*. Brocadia fulgida‐like population near the biofilm–water interface while the *Ca*. Brocadia sp. 40 population was detected throughout the biofilm. Furthermore, despite studies showing that *Ca*. Kuenenia have higher substrate affinity than *Ca*. Brocadia, and hence would be selected for at low substrate concentrations (van der Star *et al*., 2008; Oshiki *et al*., 2011), *Ca*. Brocadia fulgida and/or *Ca*. Brocadia sp. 40, rather than *Ca*. Kuenenia have dominated the anammox guild in this and other main stream PNA studies (Gilbert *et al*., 2014; Lotti *et al*., 2014a,b).

The AOB community was dominated by two populations within the *Nitrosomonas europaea/eutropha* cluster (Table 2, S3), which are commonly found in PNA reactors (Park *et al*., 2010; Vlaeminck *et al*., 2010; Pellicer‐Nàcher *et al*., 2014). Minor OTUs similar to *N. oligotropha* (sp. JL21) and *Nitrosospira multiformis* were also detected. This diverse AOB community was confirmed by FISH.

NOB were constantly present (Fig. 1) and were active during all periods, as seen by the production of nitrate (Table 1, Figure S1) and batch activity tests (Fig. 2). In particular, they had large impact on the process performance in period VI, resulting in low nitrogen removal efficiency (11%). Strategies to abate NOB include careful control of DO‐ and substrate concentrations (Perez *et al*., 2014) as well as intermittent periods of anoxic and aerated phases, either at high‐or low‐DO concentrations (Wett *et al*., 2013; Ma *et al*., 2015). Despite maintained ammonium concentrations and careful DO control, NOB could not be repressed in the MBBR biofilms. Unwanted NOB activity in main stream PNA reactors is frequently reported (De Clippeleir *et al*., 2013; Gilbert *et al*., 2014; Lotti *et al*., 2014a) and the strategy for NOB repression depending on the aggregation state of the biomass (suspended, granular, biofilm) and the ecophysiology of AOB and NOB.

The NOB consisted of *Nitrobacter*,* Nitrospira* and *Nitrotoga*. In PNA reactors, the low bulk concentrations of nitrite would select for *Nitrospira*, rather than *Nitrobacter*, due to their higher substrate affinity (Isanta *et al*., 2015). *Nitrospira* is furthermore hard to outcompete using low DO concentrations due to their high oxygen affinity (Isanta *et al*., 2015). *Nitrospira* has, in fact, been the only NOB observed in some main stream PNA reactors (De Clippeleir *et al*., 2013; Gilbert *et al*., 2014), but as shown here and elsewhere (Liu *et al*., 2012), even *Nitrobacter* can sustain at such conditions. Interestingly, the long‐term operation of the MBBR at 13°C allowed the establishment of a small *Nitrotoga* population (Table 2, S4). *Nitrotoga* has been shown to be important in activated sludge communities at 7–16°C (Lücker *et al*., 2015), but so far little is known about their ecophysiology, except for their low temperature requirements. *Nitrotoga* has, to the best of our knowledge, not been previously detected in PNA reactors and this finding may have implications for NOB suppression strategies.

In the MBBR, 43–50% of the sequences were affiliated to putative heterotrophic bacteria. Also in other studies of PNA systems, significant fractions of the microbial communities have been heterotrophs, although little is known about their composition, dynamics and roles (Chu *et al*., 2015; Gilbert *et al*., 2014; Pellicer‐Nàcher *et al*., 2014). The relative abundances of the major contributors of the heterotrophic community in the MBBR (from seven phyla) were stable in relative abundance throughout the study (Fig. 4). This implies that the decreasing COD concentrations in the reactor (Table 1) had little impact on the heterotrophic community and suggests that even heterotrophic bacteria were an integral part of the community, possibly with defined roles in the PNA biofilm. Heterotrophic bacteria may contribute to nitrogen removal via denitrification, but their competition with AOB and anammox bacteria for space and electron acceptors can also be detrimental (Kumar and Lin, 2010). Furthermore, they can utilize soluble microbial products (SMP) from the biofilm (Ni *et al*., 2012) and aid in biofilm formation (Cho *et al*., 2010). A minor fraction of the influent COD was consistently removed in the MBBR (Table 1) and batch activity tests showed anoxic nitrate uptake (Fig. 2), which suggests some denitrification. We found members of *Rhodocyclales*,* Burkholderiales*,* Rhizobiales* and *Xanthomonadales* (Fig. 4), which all are important contributors to wastewater denitrification (Baytshtok *et al*., 2009; McIlroy *et al*., 2016) and have been detected in PNA reactors treating organic‐free wastewater (Pellicer‐Nàcher *et al*., 2014; Chu *et al*., 2015), suggesting SMP utilization. SMP may also have sustained the biofilm population of the non‐denitrifying, protein degrading *Saprospirales* (Xia *et al*., 2008). *Chloroflexi* were abundant here (Figs. 3 and 4), as well as in other PNA communities (Gilbert *et al*., 2014; Chu *et al*., 2015). *Chloroflexi* can provide biofilm structural integrity (Cho *et al*., 2010) and metabolize SMP from autotrophs at both aerobic and anoxic conditions (Okabe *et al*., 2005; Kindaichi *et al*., 2012), which would explain their distribution throughout the biofilm (Fig. 5). *Phycisphaerae, Ignavibacteriales, Deinococcales* (Fig. 4) harbour bacteria with mostly undefined ecophysiologies, but their presence here and in other PNA‐ and anammox communities (Costa *et al*., 2014; Chu *et al*., 2015) suggests defined functions.

There are several possible explanations for the observed stability and maintained diversity of the microbial community at the decreasing substrate concentrations. The main anammox bacteria (*Ca*. Brocadia sp. 40 and *Ca*. Brocadia fulgida) and AOB (*Nitrosomonas europaea/eutropha*) have been detected at high relative abundances at different conditions, including a wide range of substrate concentrations (Park *et al*., 2010; Vlaeminck *et al*., 2010; Almstrand *et al*., 2014; Gilbert *et al*., 2014; Lotti *et al*., 2014a; Pellicer‐Nàcher *et al*., 2014), which indicates broad ecophysiologies and a competitiveness at all tested concentrations. Furthermore, the presence of numerous micro‐environments in the thick biofilms with gradients of substrate and electron acceptors likely promoted diversity and permitted the coexistence of competing as well as of commensal community members. As mentioned, the community had both active and non‐active bacteria; the low ribosomal content of a large fraction of the bacteria indicated inactivity (Figure S4). The protected environment in the biofilm carriers may offer a refuge site for active and inactive cells, which may slow down community changes, as would the low temperature. Although the time between subsequent test periods may have been too short for major community changes to occur, for the entire study, spanning 302 days, time was likely sufficient. In municipal wastewater, the continuous variations over time in influent composition of substrates and suspended bacteria, are factors that may affect the stability of the microbial community, but these were not addressed here. Very few studies have been performed on PNA using real main stream wastewater, and the impact of these factors is yet not valuated. Maintenance and activity of anammox and AOB populations for at least 240 days in PNA MBBRs receiving pre‐treated municipal wastewater was recently shown (Laureni *et al*., 2016), indicating that, at least for biofilm systems, the influence of these factors for the stability of the key functional populations is manageable.

In conclusion, the bacterial community in a PNA MBBR system was stable during decreasing concentrations of substrate, approaching main stream conditions. Within the guilds of AOB, anammox bacteria and NOB, composition and diversity was maintained at all tested concentrations. The composition was largely stable even for the diverse heterotrophic community, suggesting that they were an integral part of the community.

## Experimental procedures

### The pilot moving bed biofilm reactor

A 200 l MBBR was filled with biofilm carriers (Kaldnes K1) at 40% filling degree. It received reject water after anaerobic digestion from the Himmerfjärden WWTP in Stockholm, diluted with tap water. The study period (302 days) was divided into six periods with stepwise decreased influent concentrations of ammonium from 500 mg‐N l^−1^, representative of reject water, to 45 mg‐N l^−1^, representative of main stream wastewater, with concomitant decreases in nitrogen loading rate and hydraulic retention time (Table 1). The MBBR was operated at 13°C corresponding to low main stream temperatures in moderate climates.

Redox, pH and DO was measured using online sensors (Cerlic AB, Segeltorp, Sweden). Air supply was provided from the bottom of the reactor and was controlled, via the DO, by a PID controller. The temperature was monitored and controlled by a compact controller (JUMO GmbH & Co. KG, Fulda, Germany) and a cooler (JULABO GmbH, Seelbach, Germany). Mixing of the bulk water and biofilm carriers was achieved by a two‐blade stirrer (50 rpm).

For analysis of inorganic nitrogen species and COD in the influent and effluent (filtered 0.45 μm), Dr. LANGE cuvettes were used on a XION 500 Spectrophotometer (HACH LANGE GmbH, Düsseldorf, Germany).

### Batch tests for potential activity measurements

Batch tests were performed to measure potential microbial activities at 25°C. The activity measurements of AOB and NOB was based on the oxidation rate of ammonium (present in the test medium) and nitrite (formed by the AOB during the test) by measuring the oxygen uptake rate (OUR) using a DO probe (YSI 5905; YSI Inc. Yellow Springs, OH, USA). The method was adopted from Surmacz‐Górska *et al*. (1996). At the start of each measurement, ammonium was available at 100 mg N l^−1^. First, the total OUR was measured. After 5 minutes, NaClO_3_ (17 mM) was added for the inhibition of NOB. After 10 minutes, allylthiourea (43 μM) was added for the inhibition of AOB. The activity of NOB and AOB was obtained from the OUR before minus the OUR after the addition of NaClO_3_ and allylthiourea respectively. The remaining OUR, after addition of both inhibitors, is represented by endogenous respiration and substrate oxidation by heterotrophs and was not reported. The potential anammox activity was measured as the production of nitrogen gas (headspace pressure) according to Dapena‐Mora *et al*. (2007) with NH_4_^+^ and NO_2_^−^ at initial concentrations of 70 mg N l^−1^ each. Nitrate uptake rate measurements were used for measurements of potential heterotrophic denitrifying activity. The tests were performed at anoxic conditions in reject water diluted with distilled water at an initial COD concentration of 200 mg O_2_ l^−1^, with NaNO_3_ at an initial concentration of 100 mg N l^−1^. Samples for measurement of NO_3_^−^were taken every 12 minutes for 4 hours.

### DNA extraction

DNA was separately extracted from three carriers at each sampling occasion. From each carrier, 30 mg of biofilm was used for extraction using the FastDNA spin kit for soil (MP Biomedicals, Santa Ana, CA, USA) according to the manufacturers' recommendations. The concentration of the extracted DNA was measured using a NanoDrop ND‐1000 spectrophotometer (Thermo Scientific NanoDrop products, Wilmington, DE, USA).

### Quantitative PCR

qPCR was used for quantification of autotrophic nitrogen converting bacteria according to Persson *et al*. (2014). In brief, primers for the 16S rRNA gene were used to target all bacteria, anammox bacteria, *Nitrospira* and *Nitrobacter* and primers for the amoA gene were used to target AOB. The qPCR was carried out on an iQ5 (Bio‐Rad Laboratories. Inc., Hercules, CA, USA) thermal cycler using the SYBR green chemistry. Plasmid target gene inserts were used as standards. The results are presented as copy number fractions of the nitrogen converging bacteria to all bacteria, to get relative abundances. Differences in the relative abundances between periods I–VI was tested by one‐factor analysis of variance (ANOVA). Prior to ANOVA, variance homogeneity was confirmed by Levene's test. To assess whether there was a link between the reactor conditions and the relative abundance of the key microbial groups at each sampling occasion (*n* = 17), the preceding reactor concentrations of nitrogen species, COD and alkalinity was averaged over 10 days. Correlations between these average concentrations and the relative abundances were tested for using a linear model (Pearson's r).

### High throughput amplicon sequencing

PCR was carried out using the primers 515F and 806R to amplify partial V4 region sequences of the 16S rRNA gene (Caporaso *et al*., 2011) with dual indexing of the primers (Kozich *et al*., 2013). Sequencing was performed on an Illumina MiSeq (Illumina Inc., San Diego, CA, USA) using the MiSeq Reagent Kit v2 with PhiX control library spiked in at 7.5%. For details on PCR, purification, and quality control, see supporting methods. The obtained sequences were processed in Mothur (Schloss *et al*., 2009) for assembly of contigs, denoising, removal of putative chimera, alignment, classification and construction of operational taxonomic units (OTUs) at 97% taxonomic identity (Kozich *et al*., 2013). For classification with the Bayesian classifier within Mothur, the Greengenes database v. 13.8.99 (McDonald *et al*., 2012) was used at 80% confidence threshold. Prior to analysing alpha‐ and beta‐diversity, the OTU dataset was subsampled at 10 000 sequences. Raw sequence reads were deposited at the NCBI Sequence Read Archive, no. SRP059362.

### Fluorescence *in situ* hybridization and confocal laser scanning microscopy

For FISH, samples were taken in the periods II, IV and VI. The carriers were fixed in paraformaldehyde (4% w/v) for 8 h at 4°C. For FISH on biofilm suspension, two carriers form each sampling period were used. The fixed biofilm was brushed off the carriers and homogenized in PBS before storage in PBS‐ethanol (1:1) at −20°C. The biofilm suspensions (2–4 μl) were spotted on diagnostic microscope slides (8 × 6 mm diameter wells; Menzel GmbH, Braunschweig, Germany) for FISH and images were acquired from 10 random fields of view for each carrier. For FISH on biofilm cryosections, the carriers with fixed biofilm were embedded, frozen and cryosectioned in 20–25 μm thick slices which were captured on microscope slides and subjected to FISH. FISH was carried out at 46°C for 2 h for biofilm suspensions and 4 h for biofilm cryosections according to Almstrand *et al*. (2014). The probes (Table S1) were 5′ labelled with Cy3, Cy5, or Alexa 488. The relative abundances of the anammox bacteria, AOB and NOB was estimated on biofilm suspensions as the ratios of the FISH‐targeted biovolumes of the specific populations to the total bacteria (EUB338 I‐IV probe mix, Table S1) using daime 2.1 (Daims *et al*., 2006). See supporting methods for details about embedding, cryosectioning, image acquisition and image analysis.

## Conflict of Interest

None declared.

## Supporting information


**Fig. S1.** Influent and effluent concentrations of ammonium, nitrite and nitrate in the MBBR. Dashed vertical lines highlight the different periods in the study.
**Fig. S2.** Biofilm carrier from the MBBR.
**Fig. S3.** Biomass wet weight of the biofilm carriers. Dashed lines show the transition between different periods. Average values of eight carriers at each sampling occasion. Error bars show standard deviation.
**Fig. S4.** Comparison between FISH (using EUBmix probe) and staining of cells with SYTO 62.
**Table S1.** FISH probes used in the study.
**Table S2.** Diversity of the biofilm communities by high throughput amplicon sequencing. 1000 resamplings of 10 000 sequences. OTUs clustered at 97% sequence similarity.
**Table S3.** Potential autotrophic nitrogen removing OTUs clustered at 99% sequence similarity.
**Table S4.** Percentage of anammox, AOB and NOB during the experiment. Data are range of percentages over periods during the experiment (see Table 1 for time periods). FISH data are biovolume of specific probe targeted guilds in percentages of the total biovolume measured by EUB probe mix (see Table S1 for probes). FISH data are from periods II, IV and VI. High throughput amplicon sequencing data are percentages of OTUs from target group out of the total number of OTUs during periods I, IV, V and VI. qPCR data are percentages of the different target groups of the total bacteria measured by a universal primer pair. qPCR data are from periods I – VI. See Experimental Procedures for details.
**Data S1.** Supporting methods and references.
