# Supplementary material for: Community structure of partial nitritation‐anammox biofilms at decreasing substrate concentrations and low temperature
Source: Microb Biotechnol. 2016 Nov 14;10(4):761–72. doi: 10.1111/1751-7915.12435 (PMC5481546; doi:10.1111/1751-7915.12435)
Supplement: Supplementary file 1 — Fig. S1. Influent and effluent concentrations of ammonium, nitrite and nitrate in the MBBR. Dashed vertical lines highlight the different periods in the study. Fig. S2. Biofilm carrier from the MBBR. Fig. S3. Biomass wet weight of the biofilm carriers. Dashed lines show the transition between different periods. Average values of eight carriers at each sampling occasion. Error bars show standard deviation. Fig. S4. Comparison between FISH (using EUBmix probe) and staining of cells with SYTO 62. Table S1. FISH probes used in the study. Table S2. Diversity of the biofilm communities by high throughput amplicon sequencing. 1000 resamplings of 10 000 sequences. OTUs clustered at 97% sequence similarity. Table S3. Potential autotrophic nitrogen removing OTUs clustered at 99% sequence similarity. Table S4. Percentage of anammox, AOB and NOB during the experiment. Data are range of percentages over periods during the experiment (see Table 1 for time periods). FISH data are biovolume of specific probe targeted guilds in percentages of the total biovolume measured by EUB probe mix (see Table S1 for probes). FISH data are from periods II, IV and VI. High throughput amplicon sequencing data are percentages of OTUs from target group out of the total number of OTUs during periods I, IV, V and VI. qPCR data are percentages of the different target groups of the total bacteria measured by a universal primer pair. qPCR data are from periods I – VI. See Experimental Procedures for details. Data S1. Supporting methods and references. [file MBT2-10-761-s001.docx]

# Supporting material

Community structure of partial nitritation-anammox biofilms at low temperature and decreasing ammonium concentrations

Frank Persson, Carolina Suarez, Malte Hermansson, Elzbieta Plaza, Razia Sultana, Britt-Marie Wilén.

**Content:**

- Table S1
- Table S2
- Table S3
- Table S4
- Figure S1
- Figure S3
- Figure S3
- Figure S4
- Supporting methods
- Supporting references

**Table S1**. FISH probes used in the study.

* Applied with unlabeled competitor probe: 5'- GGA TGC CGT TCT TCC GTT GAG CGG -3' as described in Persson et al. 2014.

** Applied with unlabeled competitor probe(s) as defined in the reference.

**Table S2**. Diversity of the biofilm communities by high throughput amplicon sequencing. 1000 resamplings of 10 000 sequences. OTUs clustered at 97% sequence similarity.

| Sample period | Number of OTUs (S) | Inv. Simpson index |
| --- | --- | --- |
| I | 523 | 3.1 |
| III | 498 | 3.2 |
| V | 477 | 3.1 |
| VI | 519 | 3.9 |

**Table S3.** Potential autotrophic nitrogen removing OTUs clustered at 99% sequence similarity.

**Table S4.** Percentage of anammox, AOB and NOB during the experiment.

Data are range of percentages over periods during the experiment (see Table 1 for time periods). FISH data are biovolume of specific probe targeted guilds in percentages of the total biovolume measured by EUB probe mix (see Table S1 for probes). FISH data are from periods II, IV and VI. High throughput amplicon sequencing data are percentages of OTUs from target group out of the total number of OTUs during periods I, IV, V and VI. qPCR data are percentages of the different target groups of the total bacteria measured by a universal primer pair. qPCR data are from periods I – VI. See Experimental Procedures for details.

**Figure S1**. Influent and effluent concentrations of ammonium, nitrite and nitrate in the MBBR. Dashed vertical lines highlight the different periods in the study.


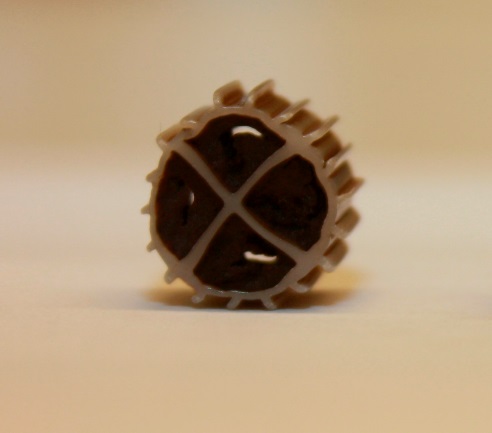


**Figure S2**. Biofilm carrier from the MBBR.

**Figure S3**. Biomass wet weight of the biofilm carriers. Dashed lines show the transition between different periods. Average values of eight carriers at each sampling occasion. Error bars show standard deviation.


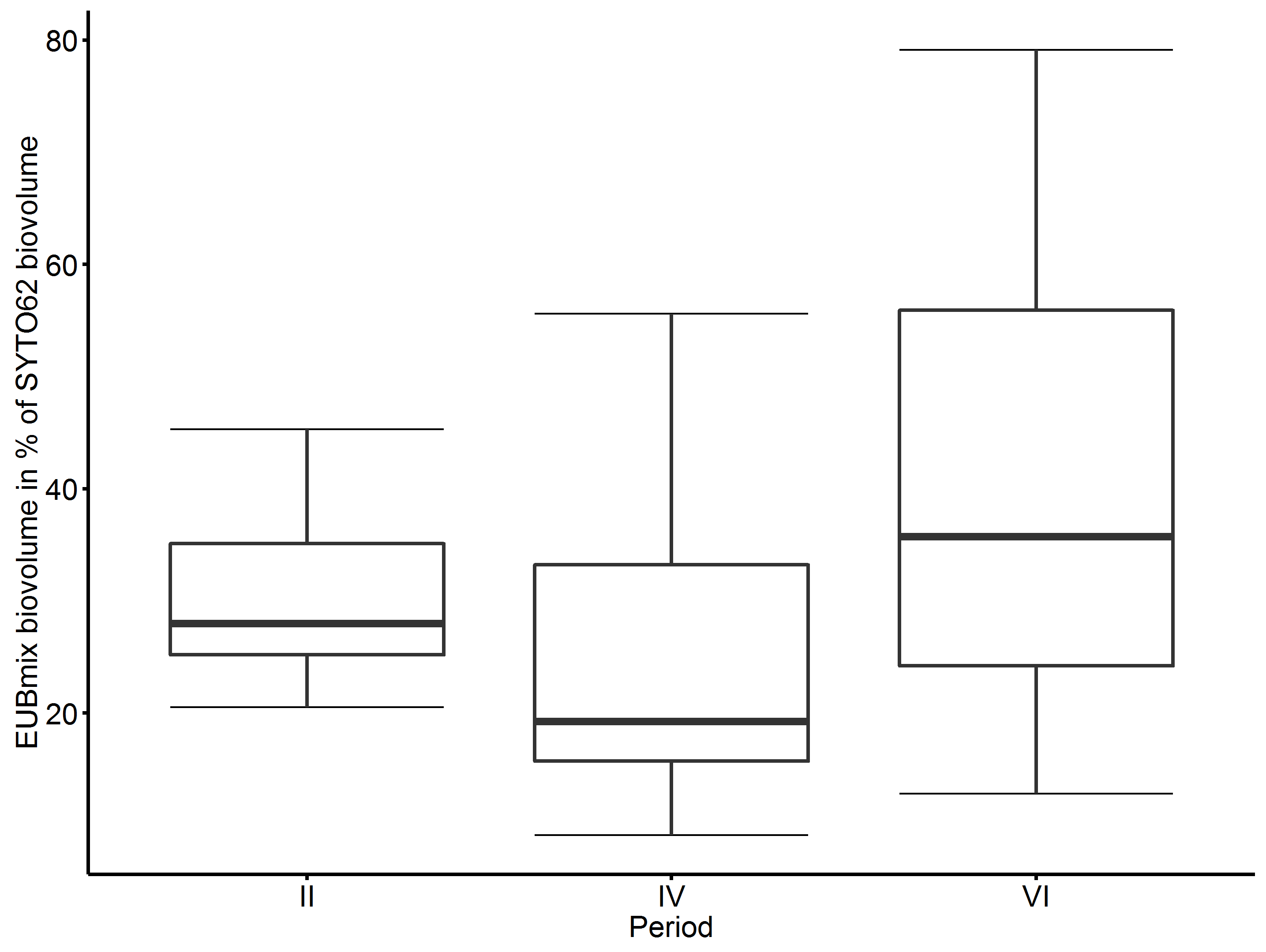


**Figure S4**. Comparison between FISH (using EUBmix probe) and staining of cells with SYTO 62.

**Supporting methods**

**Preparation for high throughput amplicon sequencing**

Prior to PCR, the three independent DNA extracts from each sampling occasion were pooled in equimolar amounts. The PCR reactions were carried out in duplicate reactions with 1 µl of target DNA, 1 µl of forward- and reverse primers (10 µM) and 17 µl of AccuPrime Pfx polymerase SuperMix (Life Technologies). The PCR program consisted of activation (95°C, 5 min); 30 cycles of denaturation (95°C, 20 s), annealing (55°C, 15 s) and elongation (68°C, 60 s); followed by final elongation (68°C, 10 min). The obtained products were quality checked by standard gel electrophoresis. The duplicate PCR products were pooled and diluted to a target concentration of 10 ng µl-1. Purification was performed using the Agencourt Ampure beads technology (Beckman Coulter). The DNA concentration of the purified products was measured using the Qubit 2.0 fluorometer (Life Technologies), the PCR products were multiplexed in equimolar amounts and the pooled PCR product was diluted with Tris-Cl (pH 8.5, 0.1% Tween20) for a final concentration of 0.6 ng µl-1, as measured by Qubit. Quality control of the pooled PCR product was performed on a TapeStation 2200 (Agilent Technologies). PhiX control library was spiked in at 7.5%. Sequencing was performed on an Illumina MiSeq using the MiSeq Reagent Kit v2.

**Cryosectioning prior to FISH**

The fixed biofilm carriers were embedded in O.C.T compound (VWR, Radnor, PA, USA) overnight at 4 °C and were subsequently frozen solid prior to taking out the embedded intact biofilm from one of the carrier compartments. This biofilm was subjected to cryosectioning in 20-25 µm thick sections using a HM550 microtome cryostat (MICROM International GmbH, Germany). The sections were captured on SuperFrost Plus Gold microscope slides (Menzel GmbH, Germany). After dehydration in an ethanol series (50%, 80% and 96% v/v), the microscope slides were stored at 20°C. Prior to FISH, a second fixation of the cryosectioned biofilm in paraformaldehyde (4%, 30 min) was performed.

**Image acquisition**

Confocal images of suspended and cryosectioned biofilms subjected to FISH were acquired with a Zeiss LSM700 (Carl Zeiss, Germany) using a 40×/1.3 plan-apochromat oil objective and laser diode lines of 488, 555 and 639 nm at an image size of 1024 × 1024 pixels, in frame mode with averaging = 4. The same pinhole size was used in all channels, equivalent to 1 AU for the Cy5 channel. A 600 nm short pass filter was used for Cy3 to reduce autofluorescence.

**Image analysis for biovolume determination**

Statistical digital image analysis was performed using daime 2.1 (Daims *et al.*, 2006) to determine biovolume fractions of target populations to total bacteria. For this, noise reduction (4 voxels) and median filtering (1 voxel) were applied and low intensity pixels, below a threshold of 50, were removed. For 2-D segmentation, biomass detection was done by thresholding using the RATS-L algorithm. Boolean operations were used in the image masks to remove signal not present in the EUB-reference channel.

**Supporting references**

Adamczyk, J., Hesselsoe, M., Iversen, N., Horn, M., Lehner, A., Nielsen, P.H., *et al.* (2003) The isotope array, a new tool that employs substrate-mediated labeling of rRNA for determination of microbial community structure and function. *Appl Environ Microbiol* **69**: 6875-6887.

Amann, R.I., Binder, B.J., Olson, R.J., Chisholm, S.W., Devereux, R. and Stahl, D.A. (1990) Combination of 16s Ribosomal-Rna-Targeted Oligonucleotide Probes with Flow-Cytometry for Analyzing Mixed Microbial-Populations. *Appl Environ Microbiol* **56**: 1919-1925.

Björnsson, L., Hugenholtz, P., Tyson, G.W. and Blackall, L.L. (2002) Filamentous Chloroflexi (green non-sulfur bacteria) are abundant in wastewater treatment processes with biological nutrient removal. *Microbiology* **148**: 2309-2318.

Daims, H., Bruhl, A., Amann, R., Schleifer, K.H. and Wagner, M. (1999) The domain-specific probe EUB338 is insufficient for the detection of all Bacteria: development and evaluation of a more comprehensive probe set. *Syst Appl Microbiol* **22**: 434-444.

Daims, H., Lucker, S., and Wagner, M. (2006) daime, a novel image analysis program for microbial ecology and biofilm research. *Environ Microbiol* **8**: 200-213.

Daims, H., Nielsen, J.L., Nielsen, P.H., Schleifer, K.H. and Wagner, M. (2001) In situ characterization of Nitrospira-like nitrite-oxidizing bacteria active in wastewater treatment plants. *Appl Environ Microbiol* **67**: 5273-5284.

Gich, F., Garcia-Gil, J. and Overmann, J. (2001) Previously unknown and phylogenetically diverse members of the green nonsulfur bacteria are indigenous to freshwater lakes. *Arch Microbiol* **177**: 1-10.

Juretschko, S., Timmermann, G., Schmid, M., Schleifer, K.H., Pommerening-Roser, A., Koops, H.P. and Wagner, M. (1998) Combined molecular and conventional analyses of nitrifying bacterium diversity in activated sludge: Nitrosococcus mobilis and Nitrospira-like bacteria as dominant populations. *Appl Environ Microbiol* **64**: 3042-3051.

Kartal, B., van Niftrik, L., Rattray, J., de Vossenberg, J.L.C.M.V., Schmid, M.C., Damste, J.S.S., *et al*. (2008) Candidatus 'Brocadia fulgida': an autofluorescent anaerobic ammonium oxidizing bacterium. *FEMS Microbiol Ecol* **63**: 46-55.

Lücker, S., Schwarz, J., Gruber-Dorninger, C., Spieck, E., Wagner, M. and Daims, H. (2015) Nitrotoga-like bacteria are previously unrecognized key nitrite oxidizers in full-scale wastewater treatment plants. *ISME J* **9**: 708-720.

Mobarry, B.K., Wagner, M., Urbain, V., Rittmann, B.E. and Stahl, D.A. (1996) Phylogenetic probes for analyzing abundance and spatial organization of nitrifying bacteria. *Appl Environ Microbiol* **62**: 2156-2162.

Persson, F., Sultana, R., Suarez, M., Hermansson, M., Plaza, E., and Wilen, B.-M. (2014) Structure and composition of biofilm communities in a moving bed biofilm reactor for nitritation-anammox at low temperatures. *Bioresource Technol* **154**: 267-273.

Schmid, M.C., Maas, B., Dapena, A., de Pas-Schoonen, K.V., de Vossenberg, J.V., Kartal, B., *et al.* (2005) Biomarkers for in situ detection of anaerobic ammonium-oxidizing (anammox) bacteria. *Appl Environ Microbiol* **71**: 1677-1684.

Schmid, M., Twachtmann, U., Klein, M., Strous, M., Juretschko, S., Jetten, M., *et al.* (2000) Molecular evidence for genus level diversity of bacteria capable of catalyzing anaerobic ammonium oxidation. *Syst Appl Microbiol* **23**: 93-106.

Wagner, M., Rath, G., Amann, R., Koops, H.P. and Schleifer, K.H. (1995) In-Situ Identification of Ammonia-Oxidizing Bacteria. *Syst Appl Microbiol* **18**: 251-264.

van Kessel, M.A.H.J., Speth, D.R., Albertsen, M., Nielsen, P.H., Op den Camp, H.J.M., Kartal, B., *et al.* (2015) Complete nitrification by a single microorganism. *Nature* 528(7583), 555-559.
